# Implementation of One Health approach in Jordan: Review and mapping of ministerial mechanisms of zoonotic disease reporting and control, and inter-sectoral collaboration

**DOI:** 10.1016/j.onehlt.2022.100406

**Published:** 2022-06-08

**Authors:** Sameeh M. Abutarbush, Alaa Hamdallah, Majid Hawawsheh, Lora Alsawalha, Nour Abu Elizz, Rachel Dodeen

**Affiliations:** a Department of Veterinary Clinical Sciences, Faculty of Veterinary Medicine, Jordan University of Science and Technology, Irbid, Jordan; b Communicable Diseases Department, Jordan Ministry of Health, Amman, Jordan; c Animal Health Department, Jordan Ministry of Agriculture, Amman, Jordan; d Jordan Country Office, World Health Organization, Amman, Jordan; e Veterinary Quarantine Department, Jordan Ministry of Agriculture, Amman, Jordan

**Keywords:** One Health, Zoonotic diseases, Jordan, Mapping, Inter-sectoral collaboration

## Abstract

**Background:**

Mapping across relevant sectors builds an understanding of a successful multi-sectoral One Health approach. This requires a review and understanding of existing national infrastructure, capacity, resources, and existing mechanisms for collaboration across sectors for addressing zoonotic diseases. The objective of this study is to review and map the existing structures of ministry of health and ministry of agriculture in relation to zoonotic diseases reporting and control, and inter-sectoral collaboration in Jordan.

**Methods:**

Jordanian ministerial infrastructures, mechanisms, legislation, responsibilities, programs, and activities related to zoonotic disease detection and reporting were reviewed. Publicly available information of national government agencies drawn from annual reports, official websites, program guidelines, advisories, minutes of meetings, and inter-ministerial communications were also reviewed. In addition, personal interviews with official, subject matter experts, and technical representatives of ministries of health and agriculture were conducted to gather, clarify, and verify data.

**Results:**

Although the current infrastructure of both Ministries is organized and well established, several gaps and challenges were identified. The regulations to judge and manage zoonotic disease notification and reporting need to be revised to become thorough and systematic between the two ministries. In addition, inter-ministerial zoonotic diseases reporting and notification between the two ministries is inconsistent, which may prevent reporting zoonotic disease in timely manner. The current reporting and surveillance system is closer to an indicator- based surveillance system which limits the ability to investigate and report new emerging zoonotic diseases. The capability to diagnose zoonotic diseases is not always present, and diagnostic tests used to confirm zoonotic diseases are not readily available or done for all diseases.

**Conclusions:**

Development of information sharing agreement between ministries, regulations and band y laws that organize and manage zoonotic disease notification and reporting in Jordan is needed. It is essential to review and modify the current reporting and surveillance systems at the two ministries to allow reporting new emerging zoonotic diseases. Capacity building in terms of zoonotic disease diagnosis remains vital for a One Health approach implementation in Jordan.

## 1 Introduction

Zoonotic diseases are the cause of death of millions of people around the world each year [1,2]. Emerging and re-emerging, recurring outbreaks of zoonotic infectious diseases, such as avian influenza, Ebola virus disease (EVD), and severe acute respiratory syndrome (SARS) highlight the need for inter-sectoral collaboration among human, animal and environment health sectors to aid in better disease prevention and control [3]. There is a growing demand internationally for a One Health (OH) approach to become the standard approach globally to combat the emerging infectious diseases and zoonotic threats. Success of OH approach is determined by inter-sectoral collaboration of various actors of complex health system and the ability to adapt to the local needs and the existing constraints of the health system, and enabling various stakeholders to collaborate without difficulties [4].

Zoonotic diseases in Jordan are prevalent and hold high public health significance. They pose a continuous threat to the public. Several zoonotic diseases have been reported or investigated while the statuses of others are still unknown. Along with other diseases, west Nile virus, MERS-CoV, brucellosis, tuberculosis, avian influenza (H5N1), rabies, salmonellosis and leishmaniasis, were all reported and investigated in Jordan [[5], [6], [7], [8], [9], [10], [11], [12], [13], [14]].

In the Jordan, the OH approach is strategically gaining importance from all stakeholders including public health professionals, policymakers, and researchers. Mapping across all relevant sectors builds an understanding of a successful multi-sectoral OH approach and this requires an understanding of existing national infrastructure, capacity and resources for addressing zoonotic diseases, and in particular, existing mechanisms for collaboration across sectors and disciplines. The review of the sector-specific infrastructures, mechanisms, legislation, responsibilities, programs, and activities related to zoonotic diseases detection and reporting is vital in order to identify the nodes of communication, coordination, and decision-making where health and veterinary sectors intersect, and to identify priorities and gaps that limit information sharing for action.

Currently in Jordan, zoonotic disease detection, reporting and control is mainly dealt with by MOH and MOA, with minimal contribution of ministry of environment. The objective of this study is to review and map the existing structures of MOH and MOA in relation to zoonotic diseases detection, reporting, prevention, control, inter-sectoral collaboration, and formal and informal links in the national Jordanian context, to identify the nodes of communication, coordination, and decision-making where health and veterinary sectors intersect, and to identify priorities and gaps that limit information sharing for action.

## 2 Materials and methods

This review was done between January and June 2021, for a period of six months. The methodology for this review and mapping was undertaken using publicly available information of national government agencies drawn from annual reports, official websites, program guidelines, advisories, minutes of meetings, and inter-ministerial communications. In addition, personal interviews with official, subject matter experts, and technical representatives of both ministries were conducted to gather, clarify, and verify data.

Current mechanisms used for zoonotic diseases detection, reporting, response, prevention and control were all reviewed. Ministerial activities, research, and surveillance related to zoonotic diseases field was explored, and the capacity for diagnosing zoonotic diseases was reviewed generally. The reporting channels for zoonotic diseases, reporting within each Ministry and between the two ministries were drawn and mapped. Available regulations for zoonotic diseases reporting and management (response, control and prevention) were also reviewed.

The obtained review and mapping was analyzed to identify gaps and sector specific needs and priorities, to better define nodes of communication and coordination as well as gaps for zoonotic diseases capacity building and reporting systems strengthening. Meetings and round table discussions were held with the presence of official, technical, and decision makers of both ministries to verify, discuss, and share the mapping results and gaps identified.

## 3 Results and discussion

### 3.1 Ministry of Health (MOH)

Jordan has one of the most modern health care infrastructures in the Middle East. The MOH was established in 1921 and regulates health matters in the Kingdom [15].

#### 3.1.1 Organizational structure of MOH in charge of zoonotic diseases

The unit in charge of zoonotic diseases at the MOH is the parasitic and zoonotic diseases department, which along with another six departments is supervised by and report to the communicable diseases directorate (CDD). The directorate reports to the Director of Primary Health Administration, which reports to the Secretary General of the Ministry, who reports to the Minister of Health [15].

#### 3.1.2 MOH laboratories

Almost all comprehensive and primary health centers in the MOH have a laboratory section that performs routine and simple diagnostic testing that does not need advanced equipment and facility. All MOH hospitals have laboratories that perform routine and advanced diagnostic techniques. Not all hospitals have the same diagnostic capabilities and collaboration is present among the different hospitals. All centers and hospitals are supported by the central laboratory that is linked to the MOH administration, directly and have the most advanced equipment needed for diagnostic techniques. Rabies testing is done at the Vaccine and Sera section within the CDD.

#### 3.1.3 Legislations of infectious diseases and zoonotic diseases at the MOH

In Jordan and as in many other countries, International Health Regulations (IHR) are applied for communicable and contagious diseases, including zoonotic ones. The revised International Health Regulations, by the World Health Assembly in May 2005, were adopted, and they entered into force in June 2007 and thus replaced the International Health Regulations of 1969. The International Health Regulations (2005), negotiated by WHO member states, lay down rules that countries must follow to assess health risks that may affect public health and in emergency situations, and to report and respond to them in a timely manner.

In 2005, the Communicable Disease Department of the MOH in Jordan has published the “Guide of Epidemiological Surveillance for Communicable Diseases” (GESCD). The guide was written by several local specialists and experts, adopting the IHR. It is written in Arabic language and is considered the only reference for applied communicable diseases legislations and regulations. The guide should be followed by the staff of all health centers, hospitals, and others in the MOH.

The guide is a soft cover, book that consists of 247 pages and has 53 chapters. The first few pages of the book include a table listing the distribution of the notification centers per governorates, Organizational Structure of the CDD, important telephone numbers within the MOH. The first chapter defines the epidemiological surveillance, its types and components. The second chapter describes the steps and process of outbreak investigation, including writing the reports. It also includes the list of notifiable communicable diseases (Group A: Diseases to be notified immediately and Group B: Diseases to be notified weekly & monthly), that require notification is stated to be done to the related health directorate. The third chapter illustrates the International Health Regulations (IHR) of 2005.

The fourth chapter provides a description for the outline adopted in the guide for each disease entity. Each chapter after chapter 4 is assigned to an individual disease. The disease outline adopted for each disease include notification group, description of the disease, the epidemiological status of the disease in Jordan, causative agent, case definition, diagnostic methods, tests and forms, reservoir, mode of transmission, incubation period, period of communicability, susceptibility and resistance, treatment, control and preventive measures, and notification forms where applicable.

The guide includes an outline for 45 communicable diseases, including zoonotic diseases.

#### 3.1.4 Zoonotic disease reporting & management mechanism

In the public and MOH sector, the index case is presented to the health centers (comprehensive, primary, or sub-centers) or hospital. When a zoonotic disease is suspected, confirmatory laboratory tests are requested and done at the involved health directorate or hospital, as applicable per the suspected disease. Positive test result from the laboratory of the health directorates are reported to the health directorate office. If the test is done at the hospital, a liaison officer is appointed to report the results to the health directorate office also. The health directorate starts an epidemiological investigation with team members as deemed necessary per case. The team consists of physicians, lab personnel, veterinarians, & others, and applies disease control & prevention procedures as per the guide of epidemiological surveillance for communicable diseases (GESCD). Veterinary team members are usually involved after the notification to the veterinary directorate as deemed necessary. The epidemiological investigation findings and results are summarized and specific notification forms are filled and sent by fax or are reported by telephone to the communicable diseases department (CDD) offices at the MOH. Once the CDD is notified, the case and data is evaluated and a decision is made to consider if what has been done at the health directorate is sufficient or to start another epidemiological investigation. If the latter decision is made, a team is formed of specialists, lab personnel, and veterinarians, as deemed necessary per the reported disease. The epidemiological investigation findings and results are evaluated and recommendations for disease management, control & prevention as per the GESCD, are sent to the reporting health directorate, hospital, veterinary directorate, and/or municipalities as deemed necessary by the CDD.

**Fig. 1:**
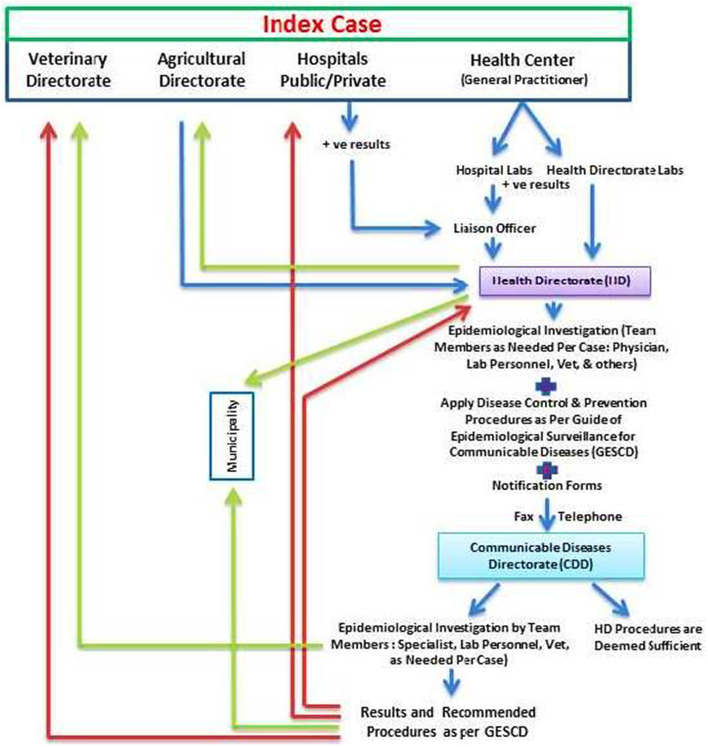
A map of the zoonotic disease reporting & management mechanism at the MOH.

In case of rabies, the disease can only be diagnosed postmortem in animals at the serology and vaccine department at the CDD. No ante mortem, specific diagnostic procedures for rabies are done on human cases suspected to be affected with rabies. However, when suspected human cases are presented, the health directorate requests the veterinary directorate to track the biting animal and quarantine it or if it is dead, to bring samples from the brain and send it to the serology and vaccine department for confirmatory rabies testing. Fig. 1 is a map of the zoonotic disease reporting & management mechanism at the MOH.

The current notification for zoonotic diseases between the MOH and MOA is done by telephone, fax, and/or official paper based inter-ministerial mail. Jordan Infectious Diseases Information System which is used for reporting and communication of communicable diseases in the MOH, including zoonotic disease, is currently being replaced by Interactive Electronic Reporting System (IERS). It is a national program of public health surveillance that is built to supports routine surveillance. It involves a case-based Integrated, disease surveillance built on existing national and international surveillance standards, guidelines and case definitions. This system generates SMS and email alerts within one hour of reporting for communicable diseases “real-time notifications”, and shares structured, anonymized data via an online framework for geospatial data visualization, generation of alerts, and automated generation of reports, with real-time analysis. It can also be used to publish online weekly epidemiological public reports.

### 3.2 Ministry of Agriculture (MOA)

The MOA was established in the era of the Emirate of East Jordan in the formation of the thirteenth ministry in 1929. It is responsible for regulating the agricultural sector, its growth and development [16].

#### 3.2.1 Organizational structure of MOA in charge of zoonotic diseases

There are two main Directorates in charge of zoonotic diseases at the MOA, Veterinary and Animal Health Directorate, and Animal Wealth Laboratories Directorate. Veterinary and Animal Health Directorate encompasses five departments, four of which deals directly with Zoonosis. These are Animal Health, Veterinary Quarantine, Poultry Health, and Slaughterhouses Departments. Animal Wealth Laboratories Directorate encompasses four departments, one of which deals mainly with Zoonosis which is Central Laboratories Department. The two veterinary directorates report to the Assistant Secretary General for Livestock, who reports to the Secretary General. The Secretary General reports the Minster of Agriculture [16].

#### 3.2.2 Legislations of infectious and zoonotic diseases

The by laws and regulations of the MOA concerning infectious diseases were reviewed and obtained from the MOA officials and were searched on the official website of the MOA [16].

Special regulations for zoonotic diseases are present for Rabies and Anthrax. The regulations for these two diseases include recommendations for disease management. The veterinarian (Public or Private) should notify the Health and Agriculture Directorates, and Governor (in case of Anthrax).

The rest of the infectious disease regulations include a list of the notifiable diseases that is adopted from the OIE and it states that the veterinarian who confirms a notifiable disease should notify the Veterinary Directorate and Animal Health Department. Moreover, there are two set of regulations that describe the recommended methods for infectious diseases spread control and prevention in general.

#### 3.2.3 MOA laboratories

Almost all Agriculture Directorates have a laboratory section that performs routine and simple diagnostic testing that does not need advanced equipment and facilities. All Agriculture Directorates are supported by the central laboratory that is linked to the MOA administration directly, and have the advanced equipment needed for diagnostic techniques including PCR, ELISA, Bacterial Culture and Sensitivity, and Rose Bengal test. However, the capability of doing ELISA and PCR for specific diseases depends on the availability of the kits, raw materials, and reagents. Rabies testing is done at the Vaccine and Sera section within the CDD, at the MOH.

#### 3.2.4 Zoonotic disease reporting & management mechanism

In the Veterinary and MOA sector, the index case is presented to the Agriculture Directorate by the farmer or public veterinarian. When a zoonotic disease is suspected, confirmatory laboratory tests are requested and done at the involved Agriculture Directorate laboratory if available. If they are not available, samples are sent to the Animal Wealth Laboratory Directorate. Positive test result from the laboratory of the Agriculture Directorates is reported directly to the Health Directorate. In addition an emergency notification form is filled and reported to the Assistant Secretary General for Livestock Affairs, who notifies the Head of the Veterinary and Animal Health Directorate (CVO). The CVO will pass the notification to the Animal Health Department, which communicate with the reporting veterinarian and farmer to ensure that measures of treatment, control and prevention are applied. Sometimes the Animal Health Department do a field visit and epidemiological investigation, and this is decided on cases by case. The applied control and prevention measures depend on veterinarian judgement and there are no specific written regulations per disease except for Anthrax and Rabies. When samples are presented to the Animal Wealth Laboratories Directorate and confirmed as positive, the Directorate notifies the Veterinary and Animal Health Directorate, which initiate the communication with the farmer, reporting veterinarian (if present) and the Agriculture Directorate. The Veterinary and Animal Health Directorate inform the CDD in the process of reporting for the zoonotic disease. When the CVO is notified by the CDD about the occurrence of a zoonotic disease, the Animal Health Department will commence a set of procedure including the communication with the farmer and may do a field visit and epidemiological investigation including taking required samples and applying the needed treatment, control and prevention protocols. When the zoonotic disease is diagnosed at the quarantine section, the Animal Health Department is then notified to do the steps described above.

**Fig. 2:**
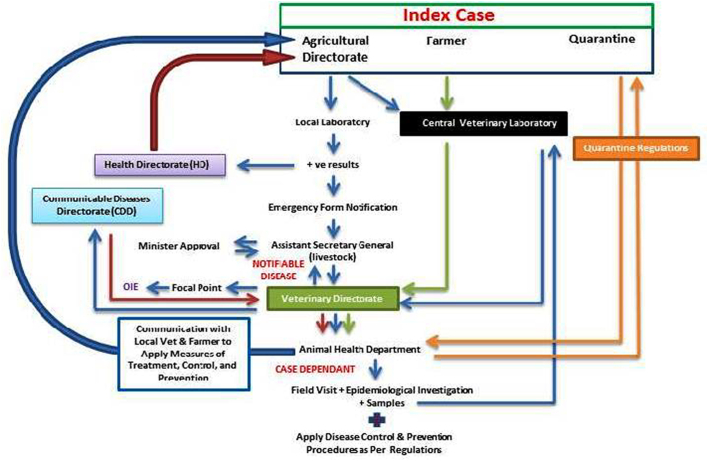
A map of the zoonotic disease reporting & management mechanism at the MOA.

In case of rabies, the disease can only be diagnosed postmortem in animals at the serology and vaccine department at the CDD. No ante mortem, specific diagnostic procedures for rabies are done on animal cases suspected to be affected with rabies. However, when suspected animal cases are presented, the Agriculture Directorate attempts to track the biting animal and quarantine it for 14 days or if it is dead (or when it is dead), bring samples from the brain and send it to the serology and vaccine department for confirmatory rabies testing. Fig. 2 is a map of the zoonotic diseases reporting & management mechanism at the MOA.

The current notification for zoonotic diseases between the MOA and MOH is done by telephone, fax, and/or official paper based inter-ministerial mail.

Jordan MOA is currently deploying the “Electronic Integrated Disease Surveillance System (EIDSS)”. EIDSS is designed to promote and support the surveillance and prevention of diseases affecting humans and animals within the concept of One World-One Health by integrating veterinary and human surveys and disease vector monitoring and facilitating compliance with the International Health Regulations (IHR) 2005. EIDSS was also developed in the framework for the Biological Threat Reduction Program (BTRP) funded and implemented by the United States Defense Threat Reduction Agency (DTRA). The System is expected to be in place and running in 2022/2023.

### 3.3 Zoonotic diseases reporting links between MOH and MOA

Zoonotic diseases reporting between the MOH and MOA occur at two levels. The first level is between the Health Directorate and Agriculture Directorate at the Governorate Level and the second one is between the CDD at MOH and the Veterinary and Animal Health Directorate at MOA.

**Fig. 3:**
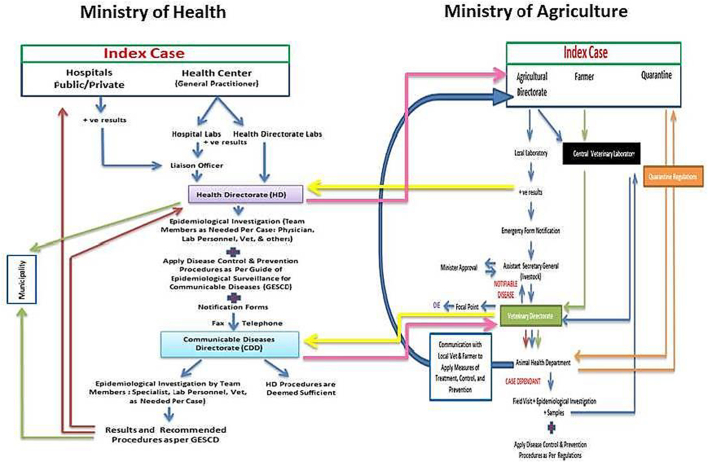
A map of the zoonotic disease reporting & management mechanism at the MOH and MOA including the connection levels (pink and yellow arrows) between the two ministries. (For interpretation of the references to colour in this figure legend, the reader is referred to the web version of this article.)

Fig. 3 is a map of the zoonotic diseases reporting & management mechanism at the MOH and MOA including the connection levels (pink and yellow arrows) between the two ministries.

### 3.4 Zoonotic diseases activities, projects and applied research at MOH and MOA

Activities related to zoonotic diseases are mainly limited to initiatives and projects funded by the international health organizations. Activities and involvement in applied research is restricted to participation in research initiated and funded by the academic institutions, Ministry of Higher Education, and funded projects from international organizations.

#### 3.4.1 Zoonotic disease prioritization

Zoonotic disease prioritization in Jordan was done in a two-day workshop, in December 2019 [17]. Participants from several organizations and sectors attended the workshop including representations from MOH, MOA, Ministry of Environment, Jordan University of Science and Technology, World Health Organization, Food and Agricultural Organization of United Nations, Jordan Civil Aviation Regulatory Commission, Eastern Mediterranean Public Health Network and Global Health Development, International Organization for Migration, and Jordan Food and Drug Administration. The main objective of the workshop was to prioritize zoonotic diseases of greatest concern for Jordan using a multi-sectoral, OH approach with equal input from representatives of human, animal (livestock and wildlife), and environmental health sectors as well as other sectors from research and higher education. During the workshop, representatives identified a list of zoonotic diseases relevant for Jordan, defined the criteria for prioritization, determined questions and weights relevant to each criterion, and developed next steps and action plans to address the priority zoonotic diseases in collaboration with OH partners [17]. The semi-quantitative selection tool developed by the U.S. Centers for Disease Control and Prevention (CDC), named CDC OH Zoonotic Diseases Prioritization Tool was adopted and five main criteria for prioritization were followed and those included disease severity, epidemiological profile, potential transmission, availability of Intervention, and Socio-economic-environmental impact [17].

The initial list of zoonotic diseases included 27 diseases, seven of which were identified as a priority by participants. These are rabies, MERS-CoV, zoonotic avian influenza, brucellosis, leishmaniasis, rickettsiosis, salmonellosis [17].

### 3.5 Gaps, challenges and limitations in mapping analysis

Several gaps and challenges were identified during this review and mapping related to deficiencies within the two ministries and sometimes due to weakness in formal links and collaboration between the two ministries and the rest of the OH partners.

The current reportingand surveillance systems at the MOH and MOA are indicator- based surveillance systems and this may limit the ability to investigate and report new emerging zoonotic diseases. Inter-ministerial zoonotic disease reporting and notification between the MOA and MOH is inconsistent. After this mapping analysis, data sharing agreement was drafted and will be signed between the two ministries based on the recommendations of this study. In addition, a user guideline for reporting zoonotic diseases in Jordan is being developed also based on the recommendation of this mapping.

The capability to diagnose zoonotic diseases by laboratory tests is not always present and diagnostic tests used to confirm zoonotic diseases are not readily available or done for all diseases, especially at the MOA. Zoonotic diseases investigation and reporting is limited to that done at the MOH and MOA and there is no involvement for the other public health (ex. Royal Medical Services), international and charity, or private sectors.

Currently, there is minimal involvement of the Ministry of Environment in zoonotic disease control only, and this is through Municipalities and mainly in disposal of dead animals. Environmental health is one of the pillars of OH approach, and the involvement of Ministry of Environment in implementation of OH approach in Jordan should be activated and more important roles must be assigned.

Zoonotic disease notification and reporting in Jordan does not appear to have specific and clear regulations to judge and manage the process. There is a lack of direct reporting between the Agriculture Directorate and Veterinary and Animal Health Directorate as well as reporting having to go through the Assistant General Secretary of the MOA. This may prevent reporting the zoonotic disease in timely manner. Additionally, there is a clear lack of governmental (MOH and MOA) applied zoonotic diseases research activity.

The analysis of this review and mapping identified sector specific needs and priorities and therefore activities can be effectively, efficiently, and sustainably established using a multi-sectoral, OH approach. In addition, duplication of effort and infrastructure will be avoided because in this mapping task, information on the full scope of national activities addressing zoonotic diseases were collected, reviewed, and analyzed in the context of the planned activity at the national level. This analysis and understanding in a country context in terms of infrastructure, stakeholders, and existing priorities will facilitate more impactful and sustainable activities and provide the baseline for monitoring and evaluation of new or strengthened activities.

Recently and based on the recommendations of the current study, MOH recognized the importance of OH approach implementation in Jordan and they established a new division for OH (Department of Zoonotic Diseases and One Health) with clear roles and responsibilities within CDD to respond to priority Zoonotic diseases in collaboration with the relevant national authorities and international organization.

## 4 Conclusions

The development of regulations and by laws that organize and manage zoonotic diseases notification and reporting to ensure consistency in reporting zoonotic diseases in Jordan is needed. Implementing information sharing agreement between the two ministries to ensure consistency and facilitate reporting zoonotic diseases will be of high value. It is essential to review and modify the current reporting and surveillance systems at the two ministries to shift them toward event- based surveillance systems to allow reporting new emerging zoonotic diseases in a timely manner. Capacity building in terms of zoonotic disease diagnosis remains vital for implementing a OH approach implementation in Jordan. Governmental (MOH and MOA) applied zoonotic disease research departments should be established with priority zoonotic diseases research topics and allocated budgets. Health systems research should be institutionalized.

## Availability of data and materials

The database generated and analyzed during the current study is available with the corresponding author.

## Funding

The project or effort depicted was or is sponsored by the United States Department of Defense, Defense Threat Reduction Agency. The content of the information does not necessarily reflect the position or the policy of the Federal Government of the United States, and no official endorsement should be inferred.

## Author statement

The authors have read and approved the revised version submitted.

## Authors' contributions

Sameeh M. Abutarbush: designing the study, methods, and writing the original draft. Alaa Hamdallah, Majid Hawawsheh, Lora Alsawalha, Nour Abuelez, and Rachel Dodeen: formal analysis, visualization, and review and editing the manuscript. All authors have approved the content, fulfill the authors' criteria, and have contributed significantly to work. All authors presented substantial contributions to this study and participated in the submitted version's correction and final approval.

## Declaration of Competing Interest

The authors declare that they have no financial and/or competing interests.

## References

[1] Lee J., McKibbin W.J., Institution Brooking (2004). Brookings Discussion Papers in International Economics.

[2] The World Bank (2012).

[3] Kelly T.R. (2020). Implementing One Health approaches to confront emerging and re-emerging zoonotic disease threats: lessons from PREDICT. One Health Outlook.

[4] Aggarwal D., Ramachandran A. (2020). One health approach to address zoonotic diseases. Indian J. Community Med..

[5] Abutarbush S., Al-Majali A. (2014). Transbound. Emerg. Dis..

[6] Sanchez M.P., Schaeffer J. (2007).

[7] Al-Natour M.Q., Abo-Shehada M.N. (2012). H5N1 influenza outbreak during March 2006 in Jordan. Sci. Res..

[8] Haddadin R.H. (2008). Poster Presented at Journées de veille sanitaire.

[9] Al-Ani F.K. (2004). Human and animal brucellosis in Jordan between 1996 and 1998: a study. Rev. Sci. Tech..

[10] Musallam I.I., Abo-Shehada M., Omar M., Guitian J. (2015). Cross-sectional study of brucellosis in Jordan: prevalence, risk factors and spatial distribution in small ruminants and cattle. Prev. Vet. Med..

[11] Rumman K.A., Sabra N.A., Bakri F., Seita A., Bassili A. (2008 Oct). Prevalence of tuberculosis suspects and their healthcare-seeking behavior in urban and rural Jordan. Am. J. Trop. Med. Hyg..

[12] Hijjawi N. (2016). Molecular diagnosis and identification of Leishmania species in Jordan from saved dry samples. Biomed. Res. Int..

[13] Tarazi Y.H., Dwekat A.F.A., Ismail Z.B. (2021). Molecular characterization of Salmonella spp. isolates from river and dam water, irrigated vegetables, livestock, and poultry manures in Jordan. Vet. World.

[14] Ababneh M.M. (2021). Longitudinal and abattoir-based surveillance of MERS-CoV in camels in Jordan, 2018-2020. Heliyon.

[15] Ministry of Health official website https://www.moh.gov.jo/.

[16] Ministry of Agriculture official website http://www.moa.gov.jo/Default/Ar.

[17] Kheirallah K.A. (2022). Prioritizing zoonotic diseases utilizing the One Health approach: Jordan’s experience. One Health.

